# Hodgkin’s Lymphoma in Older Patients: an Orphan Disease?

**DOI:** 10.4084/MJHID.2014.050

**Published:** 2014-07-01

**Authors:** Antoine Thyss, Esma Saada, Lauris Gastaud, Frédéric Peyrade, Daniel Re

**Affiliations:** Haematology Department; Centre Antoine-Lacassagne, 33 Avenue de Valombrose, 06189 Nice cedex 2, France

## Abstract

Hodgkin Lymphoma HL can be cured in the large majority of younger patients, but prognosis for older patients, especially those with advanced-stage disease, has not improved substantially. The percentage of HL patients aged over 60 ranges between 15% and 35%. A minority of them is enrolled into clinical trials. HL in the elderly have some specificities: more frequent male sex, B-symptoms, advanced stage, sub diaphragmatic presentation, higher percentage of mixed cellularity, up to 50% of advanced cases associated to EBV. Very old age (>70) and comorbidities are factor of further worsening prognosis. Like in younger patients, ABVD is the most used protocol, but treatment outcome remains much inferior with more frequent, severe and sometimes specific toxicities. Few prospective studies with specific protocols are available. The main data have been published by the Italian Lymphoma Group with the VEPEMB schedule and the German Hodgkin Study Group with the PVAG regimen. Recently, the Scotland and Newcastle Lymphoma Study Group published the SHIELD program associating a prospective phase 2 trial with VEPEMB and a prospective registration of others patients. Patients over 60y with early-stage disease received three cycles plus radiotherapy and had 81% of 3-year overall survival (OS). Those with advanced-stage disease received six cycles, with 3-year OS of 66%. The role of geriatric and comorbidity assessment in the treatment’s choice for HL in the elderly is a major challenge. The combination of loss of activities of daily living combined with the age stratification more or less 70y has been shown as a simple and effective survival model. Hopes come from promising new agents like brentuximab-vedotin (BV) a novel antibody-drug conjugate. The use of TEP to adapt the combination of chemotherapy and radiotherapy according to the metabolic response could also be way for prospective studies.

## Introduction

Both the clinical management of older patients with cancer and the design of research studies pertinent to this population remain major challenges. This statement is particularly true for Hodgkin’s Lymphoma (HL): in younger patients, HL is cured in the vast majority, but results remain disappointing for older patients, especially those with advanced-stage disease.^1^

For the treatment of HL in elderly, there is a major contrast between the relative disease frequency in older patients and the rarity of the studies specially targeted at this population. HL in older patients is not a very rare disease: in the epidemiological data from the US government, the age adjusted incidence in 2006–2008 for HL ≥ 65 represents 17.7% of all HL.^2^ Population-based epidemiological studies typically show a bimodal peak of HL incidence:^1^–^11^ the first around 30–35 years old, the second beyond 55–60.^7^

The proportion of HL patients aged > 60 ranges in the different reports between 15% and 35%;^6^,^9^,^10^,^11^,^12^ accordingly the 26.7% of biopsies of HL, collected between 2006–2011 in a database, grouping all pathologists of the department of the Alpes-Maritimes in the south of France, and including also our cancer centre, was from patients aged > 60 (unpublished data), Several recent series have specified the relative incidence of HL in the elderly and identified specific prognostic factors in this group of patients;^1^,^3^–^7^,^10^,^11^,^13^,^14^ however, only a minority of them are enrolled in clinical trials.^10^

## Specificity of HL in the Elderly

Age itself is one of the important prognostic factors in Hodgkin Lymphoma (HL) and is included in the prognostic scoring system of some of the main cooperative groups such as EORTC or Canadian-ECOG, as in the international prognostic index for advanced HL^8^ but not for the German Hodgkin Study Group, GHSG. For example, age over 50 is an unfavourable prognosis factor for the EORTC group. Patients over 60 are rarely eligible and/or included in the prospective trials of these groups. Moreover, International Prognostic Index (IPS) is the most used model for predicting prognosis in advanced-stage HL patients^8^ but it must be noted that no patient with an age > 65 was enrolled in the study. Patients aged > 60 years have a 5-year event-free survival (EFS) and overall survival (OS) rate of 30–40% and 40%–50%, respectively.^12^,^15^,^16^ These figures are significantly inferior to those reported in the adult patient.^3^ However, HL survival for all age groups increased in the last two decades, even in the age subset over 60, where no major advances in treatment efficacy were recorded, indicating an overall improvement in patient care.^1^,^3^

Several reports stressed the difference in pathogenetic mechanisms, tumour pathobiology, host-related factors, clinical presentation, symptoms and prognosis for HL affecting the elderly as compared to the adult.^4^,^5^,^13^ There are many differences between the presentation of HL in the elderly in comparison to that of the younger patients: (a) mixed cellularity is more frequent ranging from 31% to 50%;^1^,^13^ (b) 34% of cases and up to 50% of advanced cases show the presence of EBER or LMP-1 proteins in Reed-Sternberg cells^6^ (these two points are not independent); (c) B-symptoms and advanced stage are much more common than in adults.^7^ Male sex and sub-diaphragmatic presentation are also more frequent in patients over 50. Age over 70 is a factor of further worsening prognosis,^1^,^9^ as is co-morbidity.^1^ The latter seems to play an essential role in predicting treatment outcome: in a retrospective analysis, stage, B symptoms and presence of co-morbidity were independently associated with inferior survival.^12^ Several recent retrospective studies, mainly on population database*s*, have pointed out these particularities (table 1).

## Management of HL in the Elderly

There is no true consensus for treatment of HL in the elderly. After full staging, including PET –scan, geriatric assessment and evaluation of co-morbidities, the principles of treatment can be resumed as follows: for stages 1A and 2A, a short chemotherapy program of two cycles, possibly prolonged to four courses in case of poor risk features, followed by involved fields (IF) radiotherapy. For advanced stage, it is often claimed that a prolonged use of ABVD (i.e. 6 cycles) could be also given to patients in good general status, but elderly patients are often incapable to tolerate the regimen of six cycles of ABVD, particularly because of the lung toxicity induced by bleomycin. There is no consensual approach for frail patients. The number of chemotherapy cycles might be reduced depending on the result of interim PET-scans, even if data for this approach in this elderly population are lacking.

The ABVD regimen (doxorubicin, bleomycin, vinblastine, and dacarbazine) has been considered the standard front-line treatment for HL patients^17^ and remains a gold standard, especially for limited-stage HL. Obviously, treatment outcomes with ABVD in elderly patients remain inferior to those obtained in younger patients.^18^ Moreover, toxicities are more frequent and severe in older patients, and treatment dose intensity is often reduced because of toxicity and/or co-morbidity and some specific toxicities are observed, as lung toxicity of bleomycin reported as high as 46% in some series.^18^,^19^

A frequent approach in geriatric oncology or haematology to reduce toxicity of chemotherapy is the development of specific protocols that reduce dose intensity and/or omit or replace some drugs with age-associated toxicity. This approach often permits a better tolerance but is systematically associated with a reduction of response and survival, as compared to younger adult patients. For example, the role of relative dose intensity (RDI) has been questioned by the German Hodgkin Study Group (GHSG) in the elderly HL patient: a significantly lower RDI was observed in older HL patients^2^ and patients with a RDI < 65% had poorer outcomes than patients receiving higher chemotherapy doses.^9^

Taken together these data stress the concept that in elderly patients, non-HL events affect survival in a more significant way compared to younger patients.

In the late 1990s, the GHSG carried out a prospectively randomized study to compare the baseline BEACOPP regimen against COPP-ABVD, their standard regimen at the time.^20^ Between February 1993 and 1998, 75 patients aged 66 – 75 years with newly diagnosed HL in advanced stages were recruited into the HD9 trial as a separate stratum (HD9 elderly). Patients were assigned to eight alternating cycles of COPP and ABVD or eight cycles of BEACOPP in baseline doses. Radiotherapy was given to initially bulky or residual disease. In total, 68 of 75 registered patients were met assessment criteria: 26 with COPP-ABVD and 42 with BEACOPP baseline. There were no significant differences in terms of complete remission (76%), overall survival (50%), and freedom from treatment failure (FFTF) (46%) at 5 years. Two patients (8%) treated with COPP-ABVD and nine patients (21%) treated with BEACOPP died of acute toxicity. They concluded that both regimens gave limited results in elderly patients, and that baseline BAECOPP was too toxic. The use of the escalated BEACOPP, the standard regimen of the GHSG for advanced stage HL in younger patients, is excluded for patients over 60 years because of excessive toxicity.^21^

## Prospective HL Studies Specifically Designed for Older Patients

Very few prospective HL studies designed specifically for older patients are available. All studies published since 2000 are listed in Table 2. Some non-ABVD chemotherapy regimens containing different alkylating agents have been published and are also listed in Table 2.

Macpherson et al. have proposed in 2002 a new protocol called ODBEP (vincristine, doxorubicin, bleomycin, etoposide and prednisone).^22^ They compared 38 patients aged over 65, treated with this regimen, with an historical control group of 17 patients treated with MOPP/ABV-variant, a standard regimen in the 1980s. With a median survival of 43 and 39 months, and five-year overall survival (OS) of 42% and 32%, respectively, there was no statistically significant difference between the two treatments; however, ODBEP appeared to be less toxic.

The Italian Lymphoma Group Results published the results with the VEPEMB schedule (vinblastine, cyclophosphamide, prednisolone, procarbazine, etoposide, mitoxantrone and bleomycin).^12^ This study included 105 evaluable patients over 65 (mean 71 years, range 66–83), with 48 (46%) stages I–IIA, and 57 (54%) stages IIB–IV. Co-morbidity defined only as “the presence of a concomitant disease, requiring specific treatment” was observed in 39 patients (37%). The CR rate was 76%, respectively 98% for stage IIA and 58% for stage IIB–IV, and the five-year RFS rate of patients entering CR was 82%, (respectively 95% and 66%). In multivariate analysis, stage, systemic symptoms, and co-morbidity had independent value in affecting OS, DSS, and FFS. Surprisingly, despite the use of bleomycin, no pulmonary toxicity is mentioned, raising the question of this systematic evaluation.

The same regimen has been used by the Scotland and Newcastle Lymphoma study group (SNLG) in the SHIELD program^23^ for 175 patients, associating a prospective phase 2 trial of VEPEMB with 105 patients with a prospective registration study of patients not treated as part of the VEPEMB study. This trial included patients too frail to receive standard therapies and patients managed with curative intent using alternative treatment regimens, including 35 patients treated with ABVD. This somewhat complicated program was designed to recruit all patients representative of the target population in participating centers. Initial evaluation of the patients involved a formalized assessment of co-morbidity using a modified ACE-27 co-morbidity scale. Patients designated as “non-frail,” were eligible for the phase 2 VEPEMB protocol. Those designated as “frail” were eligible for entry into the registration arm of the study and were treated at their physician’s discretion. Of 103 VEPEMB patients over 60 (median age 73), 31 had early-stage disease (stage 1A/2A) and received VEPEMB three times, along with radiotherapy. The median follow-up was 36 months. Complete remission (CR) rate (intention-to-treat) was 74%, and three-year overall survival (OS) and progression free survival (PFS) were 81% and 74%, respectively. A total of 72 patients had advanced-stage disease (stage 1B/2B/3 or 4) and received VEPEMB six times. CR rate was 61% with three-year OS and PFS of 66% and 58%, respectively. Overall TRM was 7%.

Another new regimen has been developed by the GHSG in the same period in early unfavorable and advanced stages, the PVAG regimen (prednisone, vinblastine, doxorubicin, and gemcitabine).^24^ In a phase 2 study, 59 patients with HL aged 60 to 75 (median, 68 years) and with “normal organ function” and good general condition (WHO-Index ≤ 2) received 6 to 8 cycles of PVAG and additional radiotherapy if they were not in complete remission (CR) after chemotherapy. CR/CR uncertain was obtained in 46 patients (78%). The three-year estimated for OS and PFS were 58% (95% CI, 43%–71%) and 66% (95% CI, 50%–78%), respectively. WHO grade 3/4 toxicities were documented in 43 patients (73%), with one treatment-related death. These results are very close to those of their previous BACOPP regimen for survival, with less toxicity.

A study of Kolstad et al. (25) reported for the first time the results of the CHOP-21 regimen, largely used in non-Hodgkin’s lymphoma. In this single institution’s study with 29 patients aged 60 to 91, 11 stage-I/II patients received 2–4 cycle followed by radiotherapy and 18 stage-IIB/IV patients received 6–8 cycles, without radiotherapy for 13/18. Two treatment-related deaths occurred, but the complete response rate of CHOP ± radiotherapy was 93% and OS and PFS at three years were 79% and 76%, respectively. Unfortunately, these impressive results of CHOP-21 in elderly HL as not been confirmed by other groups.

## Studies Designed for Younger Patients but Including Patients over 60 Years

Other studies designed for younger patients have included patients aged over 60. They are listed in Table 3.

In 2002, the Nebraska Study Group in a retrospective comparison of ChlVPP vs. hybrid ChlVPP/ABV in 56 patients over 60 years, showed a major survival difference with a five-year OS of 30% vs. 67% respectively and conclude that adriamycin should be considered as a major component of the chemotherapy regimen in these patients.^15^ None of the usual clinical features were significant predictors of OS in patients above 60.

In the multicenter HD8 study of the German Hodgkin’s Study Group, 1064 patients with early-stage unfavorable HL were randomized to receive 4 cycles of chemotherapy (2 COPP alternated with 2 ABVD), followed by either radiotherapy (RT) of 30 Gy extended field (EF) + 10 Gy to bulky disease or 30 Gy involved field (IF) + 10 Gy to bulky disease. Of these, 89 patients (8.4%) were ≥ 60 years. Acute toxicity from RT was more pronounced in elderly patients receiving EF-RT compared with IF-RT. Freedom from treatment failure (FFTF) 64% vs. 87%, and OS 70% vs. 94% after 5 years was lower in elderly patients compared with younger patients. Elderly patients had poorer outcomes when treated with EF-RT compared with IF-RT in terms of FFTF (58% versus 70%; P = 0.034) and OS (59% versus 81%; P = 0.008). The main conclusion was that EF-RT after chemotherapy has a negative impact on elderly patients with early-stage unfavorable HL and should be avoided.^26^

The German Hodgkin Study Group (GHSG) has tested the BACOPP regimen that consists of bleomycin, doxorubicin, cyclophosphamide, vincristine, procarbazine, and prednisone.^27^ Compared with the BEACOPP baseline, a regiment largely studied by this group, etoposide was omitted, and the anthracycline dose was increased as they considered that anthracyclines might be one of the most relevant components of chemotherapy for HL in older patients**.** Sixty five patients with early unfavorable or advanced stage HL, aged between 60 and 75 years, were enrolled in this phase 2 trial. Treatment consisted of 6 to eight cycles of BACOPP. Residual tumor masses were irradiated. Of 60 eligible patients 51 (85%) achieved CR/CRu. With a median observation time of 33 months, 18 patients died (30%), including 7 treatment-associated deaths. Grade 3/4 toxicities occurred in 52 (87%) of patients. The PFS and OS rates for all patients at 3 years were 60% (95% CI: 46%–73%), and 71% (95% CI: 59%–83%) respectively. Thus, they considered this BACOPP regimen as active but compromised by a high toxicity in older HL patients.

The U.S. intergroup trial E2496 comparing ABVD vs. Stanford V (S.V) regimen has included 45 patients ≥ 60 years treated with ABVD (23 cases) or S.V (22 cases).^18^ Adjunctive radiotherapy was delivered in 8.7% of ABVD patients and 43% S.V patients with different criteria between these two groups. In these patients, 5-year EFS and of 48% and 58%, respectively, were much inferior to those obtained in younger patients (74% and 90%, respectively). Moreover, in this trial, Bleomycin lung toxicity was 24%, mainly observed with ABVD (91%), and the death rate due to Bleomycin toxicity was 18%. The overall treatment-related mortality was 9% for older patients compared to 0.3% for patients aged <60 years. Overall, the death incidence without progression at two and five years were respectively 13% and 22% for elderly patients, and 2% and 9% for younger patients (p<.0001). These results stressed that both ABVD and S.V regimens are toxic and difficult to use for patients aged above 60 and indicated the necessity of a precise evaluation and selection of patients before the choice of treatment.

Böll et al.^2^ reported in 2013, for the GHSG, 117 cases of patients aged from 60 to 75 years (median 65) in a cohort of 1299 patients, included in the HD10 and HD11 trials, and treated with 4 x ABVD for limited HL (stage I or II). Despite the selection of a favourable subgroup, the dose intensity delivered was reduced and only 59% of the patients achieved a relative dose intensity >80% compared with 85% for younger patients. In 14%, major protocol violations occurred, mainly because of excessive toxicity. Toxicities of WHO grade 3 or 4 occurred in 68%, with 5 % of treatment related mortality. The author conclude: “in patients ≥ 60 years with HL, four cycles of ABVD are associated with substantial dose reduction, treatment delay, toxicity and treatment-related mortality.”

The escalated BEACOPP, which is the standard protocol for advanced-stage HL for the GHSG and is much more toxic that ABVD, could not be proposed in patients aged over 60y : in their HD9 study the treatment related mortality (TRM) rate was high (14.3%) in patients over 60 years and neutropenic infections were the main cause of TRM^21^

## Role of Geriatric and Comorbidity Assessment in the Treatment Choice for HL in the Elderly

While definitions of the elderly patient remain vague, one can describe the ageing process as a gradual loss of adaptation to stress due to a diminution of the functional reserves of different organs. The process is variable from one individual to another and thus, the mean life expectancy of a 70 year-old patient varies from 6.7 to 18 years, and that of an 80-year old patient from 3.3 to 10.8 years. In addition to chronic pathologies (co-morbidities), geriatric syndromes (dementia, incontinence, under-nutrition, etc.) further complicate medical management. Clinical studies regularly exclude patients who are very old, frail or suffering from co-morbidities, which makes the results difficult to apply to these patients.

The clinician thus confronted a dual dilemma: should he treat with a proven but unpredictable risk of hyper toxicity, or undertreat a potentially curable patient and waste an opportunity. A recent review point out this dilemma in hematologic malignancies^28^ but data are very scarce for HL. A range of techniques have been advanced for assessing geriatric patients with a view to singling out those capable of receiving a potentially toxic dose of chemotherapy. The first step is to identify those most likely to benefit from comprehensive geriatric assessment (CGA). This method involves evaluating comorbidities, physical and psychic autonomy, nutritional and cognitive status, level of depression, mobility and balance. Unfortunately, the process is long, costly, complex and difficult to use in clinical units in the absence of a dedicated geriatrician. Other, simpler methods have been suggested but remain open to criticism and with contradictory results.

Recently, Soubeyran et al. reported interesting results of the Oncodage study in 1688 patients with solid tumors. They validated the G8 score, a relatively simple instrument to detect patients presenting at least one abnormal geriatric test among the following: Activity of Daily Living (ADL), Instrumental Activity of Daily Living (IADL), MMS, Geriatric Depression Scale 15 (GDS-15questions), Mini Nutritional Assessment (MNA), Timed Get up and Go, and evaluation of co-morbidities.^29^ Although constructed from patients with solid tumors, the G8 test appears to be useable in malignant hematology. In non-Hodgkin’s lymphoma**,** the GELA group reported a prospective series of 150 DLBCL patients over 80 years treated with low doses of CHOP and full dose of rituximab. A good PS non-bulky disease (<10 cm), albumin >35 g/l, extra-nodal sites <2, and high ADL level were associated with improved overall survival (OS) but in multivariate analysis, only albumin levels superior to 35g/l were associated with longer OS.^30^

In HL the only prospective study including a co-morbidity assessment in a patient over 60 years seems to be the SHIELD study, previously described:^23^ this study seems to be more applicable to everyday life, and serves more as a reflection than a tool for the choice of treatment for an individual patient. In their retrospective study, Evens et al. showed that the combination of loss of activities of daily living and the age stratification of about 70 years gives a simple survival model in elderly HL.^1^ Several series have shown that the presence of co-morbidities is independently prognostic.^12^,^31^,^32^ Further trials should be designed to permit some flexibility to modify or adapt the drug dosing or the regimen, on the basis of firm objective criteria to avoid prohibitive toxicities. These trials may be able to optimize the duration of treatment to the response according to PET response (cfr. infra).

## New Drugs

Brentuximab Vedotin (BV) is a promising novel antibody-drug conjugate targeting CD30 linked to a potent synthetic antitubulin chemotherapeutic agent, monomethyl auristatin E (MME). BV acts as a cell cycle-specific agent, like Vinca alkaloid drugs, and induces G2/M-phase growth arrest.^33^ A multicenter phase II trial of BV in HL patients, recurring after ASCT, demonstrated an overall response rate (ORR) of 75% and a complete response (CR) rate of 34% with a median duration of 20.5 months.^34^ The most common treatment-related adverse event was peripheral sensory neuropathy, recorded in 42% of patients, but with complete resolution after discontinuation of treatment in 50% of patients. A very similar incidence of grade 2 or 3 peripheral polyneuropathy (52%) and symptom resolution (54%) was reported by Gopal et al. in a small series (N=25) of patients treated with BV for recurring HL after allogeneic bone marrow transplantation.^35^ A higher rate of peripheral neuropathy was observed with weekly BV treatment versus treatment delivered every 3 weeks.^36^,^37^

No association of more frequent or severe neuropathy with old age was reported in any study, while a pre-existing neuropathy was found in more than half of cases of neurotoxicity. The second most frequent toxicity of grade 3 or more was haematological: anaemia (6–20%) neutropenia (20% in both series), and thrombocytopenia (8%). A higher rate of peripheral neuropathy was observed with weekly BV treatment versus treatment delivered every 3 weeks.^36^,^37^

Bendamustine hydrochloride (Be) combines the antimetabolite activity of the purine-analogue structure with the alkylating property of the nitrogen mustard group. It is not a “new” drug, but its use in HL is recent. Because of its distinctive activity profile, Be may show limited cross resistance with agents usually employed for upfront and salvage treatments, and, therefore, it has been proposed for the treatment of relapsing/resistant HL. In two small phase II studies, enrolling nearly 70 patients aged 20–75, previously treated with 3–8 lines of chemotherapy (including autologous or allogeneic stem cell transplant), the ORR and CR rate, after a mean of 3.8 cycles of Bendamustine, administered intravenously at the dose of 120 mg/m^2^ on day 1 and 2 every 28 days, were, respectively, 50–53% and 29–33%. The mean duration of response was 5 and 5.7 months.^38^,^39^ Corazzelli et al., in a third phase II study reported similar results with 6–8 courses of Be at 70–120 mg/m^2^ in 41 relapsed or refractory HL patients after 1–8 lines of chemotherapy, aged 18–85 years. The ORR and CR rate were 58% and 31%, respectively.^40^ Despite being relatively short PFS, these rates of response in such heavily pre-treated patients are encouraging.

The feasibility of Be treatment in patients aged 70 or more has been explored more frequently in Chronic Lymphocytic Leukemia (CLL). In a large retrospective study of 465 CLL patients treated with Bendamustine alone or in combination with rituximab, 91 were older than 70.^41^ The most frequently administered dose and schedule was 76–98 mg/m^2^, twice per cycle every 21 days. Nearly 40% of patients experienced no cycle delay, grade 3–4 blood/bone marrow AE occurred in 18.8% and 25.3%, respectively. In a multicenter phase II clinical trial from the German CLL Study Group, among 117 previously untreated CLL patients aged 34–78 years, 25.6% were aged > 70.^42^ Be schedule was 90 mg/m2 on days 1 and 2, every 28 days, associated to Rituximab. Grade 3 or 4 adverse events for neutropenia, thrombocytopenia, and anaemia were documented in 19.7%, 22.2%, and 19.7% of patients, respectively. However, in both reports, the myelosuppression, due to bone marrow invasion by disease in all patients, and the association with Rituximab could have negatively affected the haematological toxicity. Finally, in a phase II trial in a small cohort of 20 elderly and frail patients affected by Diffuse Large B-Cell Lymphoma (DLBCL), with a mean age of 72 (51–86), Be was administered at a dose of 90 mg/m^2^ on days 1 and 2 in association with Rituximab on day 0, every 28 days.^43^ The relative dose intensity (RDI) was 100%. Granulocyte colony stimulating factor (GCS-F) was administered in only two patients and only three grade 3 WHO (1 leukopenia and 2 anaemia) and one grade 4 (thrombocytopenia) toxicities occurred.

Taken together, all these data makes Be an interesting candidate for use in combination for treatment of HL in elderly.

## Other New Agents

Some other drugs are currently being studied and could potentially be used in the treatment of HL in the elderly because of a reduce toxicity compared to the usual chemotherapy regimen. Some epigenetic agents as HDACS inhibitors have shown encouraging results: for example, panobinostat, in a phase 2 trial, showed an ORR of 27%.^44^ Lenalidomide an immune-modulatory agent with anti-angiogenic properties demonstrated a modest single agent activity, but may also be used in combination.^44^,^45^

## Proposal for New Approaches

As the “gold standard” for younger patient, represented by ABVD, is more toxic and difficult to use with sufficient dose-intensity and efficacy in the elderly, there is room for new approaches in the treatment of HL in the elderly.

## Treatment Adapted to the Positron Emission Tomography (PET)

### Response

The early complete metabolic response, evaluated by PET after two cycles of ABVD, has been demonstrated to be the most important prognostic factor in 260 patients with advanced stage HL.^46^ Many protocols are testing the possibility of shortening chemotherapy and/or omitting radiotherapy in adult patients, especially when in early favourable stage. In the elderly, this criterion could be used to propose chemotherapy of shorter duration, to avoid unnecessary toxicities. This strategy seems to be particularly attractive in the limited stages (I or II) in which radiotherapy can be use thereafter without excessive toxicity, even in the population of older patients. The major limitation of this strategy is that most elderly patients with HL have advanced stage,^7^ reducing the indications of radiotherapy.

### Combination of BV with other chemotherapy compounds

As BV is the only truly new drug with a major activity in HL without unexpected toxicities in the elderly, its use in a first line combination should be tested on elderly HL patients. Several reasons could account for the synergistic activity of BV with other antineoplastic drugs. First, differences between the cell types targeted by each class of compounds may account for improved activity. BV action is targeted against cells expressing CD30. CD 30 is found in Hodgkin’s and Reed Sternberg (HRS) cells, which typically comprise only 0.1–1% of the total cell population within HL lesions.^47^ Chemotherapeutic agents, in addition to tumour cells, also target proliferating stromal cell infiltrates, which constitute the majority of the cell populations within HL lesions. By contrast, the cytotoxic effect of free MMAE diffused from CD30+ malignant cells on bystander cells may in part account for the significant antitumor activity of BV.^48^ Secondly, differences in the mechanism of action between MME-based ADC and chemotherapeutic compounds may help to explain the increase in efficacy.

Frontline treatment of advanced-stage HL, with BV at the dose of 1.2 mg/Kg in association with ABVD (Doxorubicin, Vinblastine, Dacarbazine) or AVD, proved very active in a phase I multicentre trial, achieving respectively 95 and 96% of CR.^49^ The most frequently reported (WHO grade ≥ 3) adverse effects were neutropenia and peripheral sensory neuropathy present in 44% of the ABVD cohort, with 2 toxic deaths.^49^ These results with BV plus AVD are promising, but it is possible that this combination will remain too toxic for elderly patients.

Be seems to be a good candidate for such a combination as it have a non-cross-resistant mechanism of action compared to BV combining the antimetabolite activity of the purine-analogue structure with the alkylating property of the nitrogen mustard group. In addition to primary effects on tumour cells, Be has shown a peculiar activity in HL by depleting the neoplastic microenvironment of tumour-supporting T- and B-lymphocytes.^50^,^51^ This drug is already widely used in patients over 70 with CL or low grade lymphoma, and toxicity is easily manageable without dose reduction in case of renal insufficiency.^52^

## Conclusions

Existing data does not define an optimal first line treatment for elderly HL patients. A comparison of existing trials is almost impossible because of the small number of patients included in the few published trials, lack of patient stratification (based on co-morbidities or age cohorts, for example), heterogeneous documentation of side effects such as lung toxicity, and varying endpoints chosen to assess efficacy of treatment protocols.

Patient selection will be crucial for an effective treatment strategy that could be modified based on interim PET imaging. In the absence of clinical data, it is necessary to individualize treatment decisions based on pre-treatment assessment scales and PET imaging. Based on our clinical experience, it is of utmost importance to monitor patients closely while on treatment in order to detect and control severe treatment related complications.

## Figures and Tables

**Table 1:** Retrospective studies of elderly patients with HL.

Author	Type of study	Population	Main Results	Ref
Stark GL 2002	population based approach Northen England	102 pts ≥ 60y	in patients ≥ 70 y : 5y DFS: early stage 36 m, advanced 14 m poorer survival for EBV+	8
Engert A (2005)	Older patient from GHSG studies	372 pts ≥ 60y	poorer risk profil, more severe TR toxicity, higher motality during treatment, lower dose intensity vs younger patients	13
Brenner H (2008)	SEER data USA survival 1980–82 vs 2007–04	_	age specific survival: absence of improvement in patient ≥ 60y : 10 y relative survival of 44,9 %	55
Björkholm M (2011)	Population data Sweden 1970–2006	_	proportion of elderly pts : 21% > 65y ; 5% ≥ 80y	57
Evens AM (2012)	Retrospective multicentre	94 pts (60 –89y)	age> 70 and loss of activities of dayly living only prognostic factor	56

**Table 2:** Recent studies with specific regimen for patients over 60 years with HL.

Study	Nb pts	Age	Protocol	Response	Survival	Toxicity	Comments	Ref
Macpherson (2002)	38	> 65 med 72	ODBEP	NR	5y OS 42%	no toxic death	radiotherapy for bulky med.	53
Levis (2004)	105	> 65 mean 71	VEPEMB	CR IIA 98% IIB–IV 58%	5y RFS IIA 95 % IIB–IV 61%	TRM 2%	pronostic value of “comorbidity”	48
Kolstad (2007)	29	≥ 60 med 71	CHOP 21 stage I–IIA 2–4+Rt stage IIB–III 6–8 + RT	CR 93%	stage I–IIA 3y OS 91% 3y PFS 82% stage IIB–IV 3y OS 67% 3y PFS 72%	2 tox. deaths	55% with comorbidities	18
Halbsguth (2010)	60	60–75 med 68	BACOPP 6–8 cycles	CR/CRU 85 %	3Y PFS 60% 3Y OS 71%	TRM 12% grade 3–4 87 %	radiotherapy for “residual mass”	50
Böll (2011)	59	60–75 med 66,7	PVAG	CR/CRU 78%	3Y PFS 58 % 3Y OS 66%	1 tox. death grade 3–4 75 %	radiotherapy for “residual mass”	49
Proctor (2012)	103	> 60 med 73	VEPEMB stage I–IIA 3+Rt stage IIB–III 6+ RT	stage I–IIA 74% CR stage IIB–IV 61% CR	stage I–IIA 3y OS 81% 3y PFS 74% stage IIB–IV 3y OS 66% 3y PFS 58%	TRM 7%	non frail patients only	46

**Table 3:** Data for patients over 60y in studies designed for younger patients.

Study	Nb pts	Age	Protocole	Response	Survival	Toxicity	Comments	Ref
Weekes (2002)	56	≥60	ChlVPP or ChlVPP/ABV	PR+CR 84%	5y OS 30% or 67%	NR	age only pronostic factor for pts >60	54
Ballova (2005)	68	66–75 Advanced	COPP/ABVD vs BEACOPP - baseline	CR 76%	5y FFTF 55% vs 74%	TRM 2% vs 8%	RT to bulky and residual mass(26%)	51
Klimm (2007)	89	60 (med 65) early unfavorable	COPP/ABVD + EFRT/IFRT	CR/CRU 93,5%	5y FFTF 64% vs 70%	2% of TRM related to EFRT	negative impact of EFRT vs IFRT	55
Evens (2013)	45	≥60	ABVD x 6–8 vs S.V x 12 w	CR/CRU ABVD 65% S.V 62%	3y OS 73% 3y FFS 56%	TRM 9%	24% lung tox. RT in 8,7% ABVD pts 43 % S.V pts	16
Böll (2013)	117	65–70 med 65 stage I–IIA	ABVD x 4 IFRT 20–30 gy	CR 89%	5y PFS 75%	TRM 5% grade 3–4 68%	GHSG H10–H11 studies	45
